# PPP Sliding Window Algorithm and Its Application in Deformation Monitoring

**DOI:** 10.1038/srep26497

**Published:** 2016-05-31

**Authors:** Weiwei Song, Rui Zhang, Yibin Yao, Yanyan Liu, Yuming Hu

**Affiliations:** 1 College of Natural Resource and Environment, South China Agricultural University, Guangzhou, 510642, China; 2 Research Center of GNSS, Wuhan University, Wuhan, 430079, China; 3 School of Geodesy and Geomatics, Wuhan University, Wuhan, 430079, China; 4 Shenzhen Key Laboratory of Spatial Smart Sensing and Services, College of Civil Engineering, Shenzhen University, Shenzhen 518060, China; 5 Guangdong Province Key Laboratory for Land use and consolidation, Guangzhou, 510642, China; 6 Guangdong Province Engineering Research Center for Land Information Technology, Guangzhou, 510642, China; 7 Key Laboratory of the Ministry of Land and Resources for Construction Land Transformation,Guangzhou, 510642, China

## Abstract

Compared with the double-difference relative positioning method, the precise point positioning (PPP) algorithm can avoid the selection of a static reference station and directly measure the three-dimensional position changes at the observation site and exhibit superiority in a variety of deformation monitoring applications. However, because of the influence of various observing errors, the accuracy of PPP is generally at the cm-dm level, which cannot meet the requirements needed for high precision deformation monitoring. For most of the monitoring applications, the observation stations maintain stationary, which can be provided as a priori constraint information. In this paper, a new PPP algorithm based on a sliding window was proposed to improve the positioning accuracy. Firstly, data from IGS tracking station was processed using both traditional and new PPP algorithm; the results showed that the new algorithm can effectively improve positioning accuracy, especially for the elevation direction. Then, an earthquake simulation platform was used to simulate an earthquake event; the results illustrated that the new algorithm can effectively detect the vibrations change of a reference station during an earthquake. At last, the observed Wenchuan earthquake experimental results showed that the new algorithm was feasible to monitor the real earthquakes and provide early-warning alerts.

Natural hazards, such as earthquakes, landslides, subsidence and avalanches are crucial, but do not occur without warnings^1^. Through multiple deformation monitoring methods on the deformable objects, abnormal behavior can be detected to warn of imminent hazardous^2^,^3^. However, for large seismic events, it is difficult for traditional seismic instruments to produce accurate and reliable displacements because of the saturation of broadband seismometers and problematic integration of strong-motion data into displacements due to sensor rotation and tilt^4^,^5^. During the last decades, Global Navigation Satellite Systems (GNSS) have emerged as one of the most effective ways to monitor instantaneous crustal movement. GNSS can observe displacements directly, making it particularly valuable in case of large earthquakes^6^,^7^,^8^. Recently, high precision real-time satellite orbit and clock products are already available from a number of IGS (International GNSS Service) analysis centers, which will further promote GNSS real-time deformation monitoring applications^9^.

There are two main high-precision GNSS processing approaches. One is the network positioning algorithm based on double-difference model, but during the processing one station must be fixed or at least tightly constrained to a known position, which is not fit for the earthquake monitoring^10^. The other is the PPP algorithm, which can be processed in a single station, avoiding the stationary reference station selection problem^11^,^12^. However, due to the effects of various errors, PPP accuracy is generally at cm-dm-level which cannot meet the accuracy requirements in earthquake monitoring. A variety of methods have been proposed to further improve PPP positioning accuracy. One is to establish temporal and spatial model for an individual observation station. Using the spatial correlation between stations, the common error such as ionospheric delay can be reduced effectively, while the sidereal day features helps to be less affected by environmental impact errors^13^. Recently, ambiguity resolution techniques dedicated to a single station have been developed successfully to further improve the positioning accuracy of PPP^14^,^15^,^16^. However, because of different observation environments and satellite products, there may exist a risk of fixing error^17^.

In this paper, we presented a new PPP algorithm to improve dynamic PPP precision. During most of the coseismal deformation monitoring, the reference stations are static; under this assumption, a combination algorithm of kinematic PPP and static PPP based on sliding window was put forward. The new algorithm was validated with multiple GNSS observation data, including IGS tracking stations data, simulated earthquake experiment data and measured seismic data. The experimental results showed that, compared with the traditional algorithm, the new method can improves dynamic PPP precision dramatically, especially for the vertical direction, making it applicable for earthquake monitoring applications.

## A New PPP algorithm based on sliding window

During the PPP processing, the observational errors can’t be eliminated by differencing method. Most of them are removed by parameter estimation or empirical models and so on^18^,^19^. The basic mathematical model of PPP is as follows:


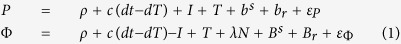


where *P* is the pseudo-range observation; Φ is the carrier-phase observation; *ρ* is the undifferenced ranges of the satellite-receiver; *c* (m/s) is the speed of light in a vacuum; *dt* is the satellite clock bias; *dT* is the receiver clock bias; *I* is the ionosphere delays; *T* is the troposphere delays; *λ* (m)is the signal wavelength; *N* is the carrier-phase ambiguity; *b*^*s*^ is the satellite part pseudo-range inner channel bias (ICB); *b*_*r*_ is the receiver part pseudo-range ICB; *B*^*s*^ is the satellite part carrier-phase ICB; *B*_*r*_ is the receiver part carrier-phase ICB; *ε*_*P*_ is the uncorrected systematic and random errors of pseudo-range; *ε*_Φ_ is the uncorrected systematic and random errors of carrier-phase.

In accordance with an object’s motion state, PPP can be divided into two different modes: static and dynamic. In the static mode, the observation stations are considered to be in a quiescent state during the observation arcs; all the observed epochs are used to calculate one coordinate. So the impacts of the observational errors can be reduced, and accurate float ambiguities are easily obtained. However, in the dynamic mode, due to the strong randomness motions, it is difficult to establish an accurate motion state equation and build the relevance of station coordinates during the observational time for a moving object. Therefore, the coordinate parameters need to be estimated for each epoch, which causes more parameters and lower accuracy. However, during the deformation monitoring, the reference stations are generally static or with small deformations before the occurrence of a disaster. According to this, we designed a combinational algorithm of dynamic and static PPP. The new algorithm can overcome the long period static demands of static PPP and the low accuracy of dynamic PPP using a positional results sliding window approach.

**Figure 1:**
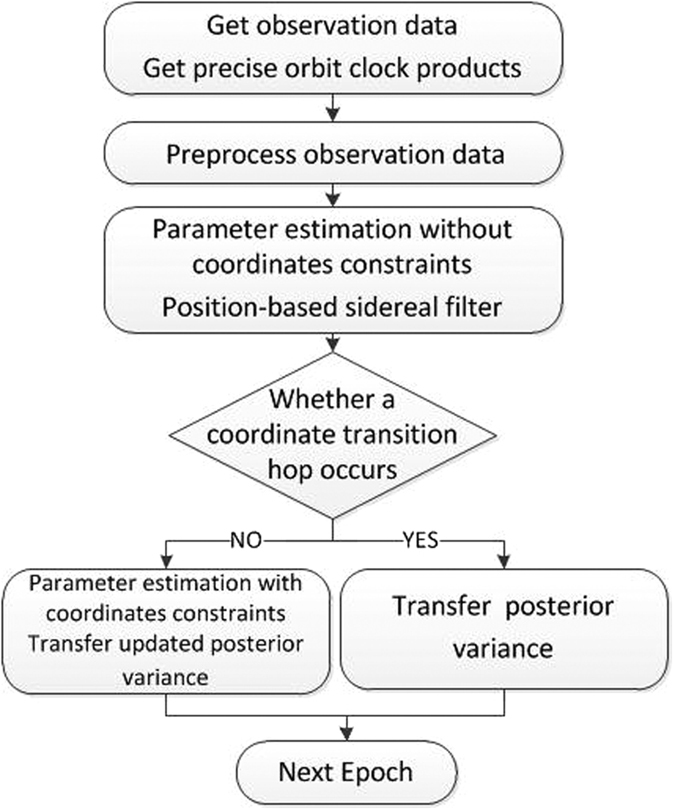
PPP calculation flow based on a sliding window.

For each epoch, firstly the coordinate constraints are relaxed, and the new observations are processed with the priori ambiguity parameters by a Kalman filter. In addition, the station coordinate time series are calibrated to mitigate multipath error, which approximately repeats every sidereal day. This calibration is computed using a position-based sidereal filter, by stacking the coordinate time series from the previous day, shifting each series by 4 min per day. Then a sliding window is used to analyze the updated coordinates’ time series changes. In order to ensure the accuracy and minimize the effect of possible small deformation at the same time, the length of sliding window is set as 6 hours. If any one of the three orientation calculation results is over triple mean square error, a coordinate transition hop judged to occur. Then the current epoch will be marked, and the coordinate constraints will be released since next epoch; if not, the observations are reprocessed with coordinate constraints and the updated ambiguity variance information is passed onward to the next epoch. Figure 1 shows the PPP calculation flow based on a sliding window.

## Experiments

### Simulation Dynamic Experiment

**Figure 2:**
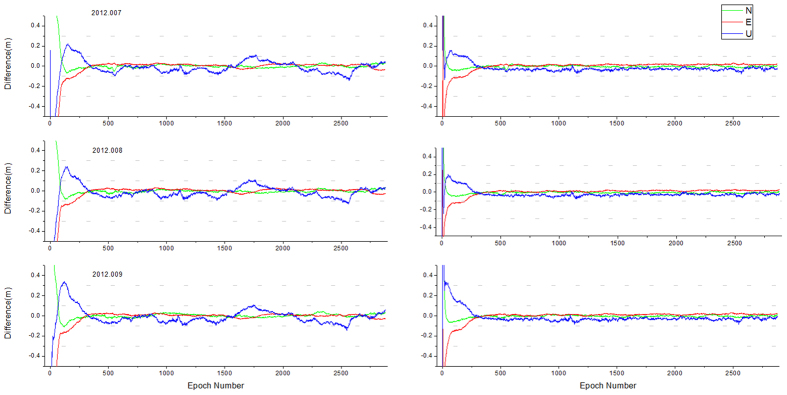
Time sequences of the simulation dynamic experiments at BJFS station.

The IGS tracking station BJFS was selected to carry out the simulation dynamic experiment. The observation data from Day 007 to 009 of 2012 was chosen, and the observation interval was 30s. The final orbit and clock products from IGS were used to correct the satellite orbit and clock error during the experiment. For all three days, the data were processed by both traditional algorithm and the new sliding window-based algorithm. The reference coordinates for comparison were the weeks’ solutions from ITRF. Figure 2 showed the time sequences of the position results, the left was the traditional algorithm and the right was the new algorithm, respectively.

As can be seen from Fig. 2, the position results of each day showed high repeatability. For the traditional algorithm, the position accuracy was about 5 cm for the horizontal and 10 cm for the vertical after convergence, and the position results showed a consistent trend for all three days. This might be a result of obvious changes of satellite distribution or the strong m-path effects, which would need further analysis. The results of new sliding window-based PPP algorithm were much more continuous and smooth, with deviation within 3 cm for horizontal and 5 cm for the vertical. Table 1 showed the statistics results of DoY 007, 2012.

As can be seen in the table, compared with the traditional algorithm, the new algorithm improved the accuracy for three directions, especially for the vertical direction, where the STD value decreased from 4.8 cm to 1.4 cm, which was close to the horizontal accuracy. It should be noted that there was a −2.9 cm systematic bias for the vertical direction, which might be caused by some incomplete corrections (such as ionosphere higher order term, antenna phase center model, etc.). However, during coseismal monitoring, the time series of calculation results were analyzed to determine whether a coordinate transition hop occurred, so the systematic bias would have no impact on the determination.

### Simulation Earthquake Experiment

**Figure 3:**
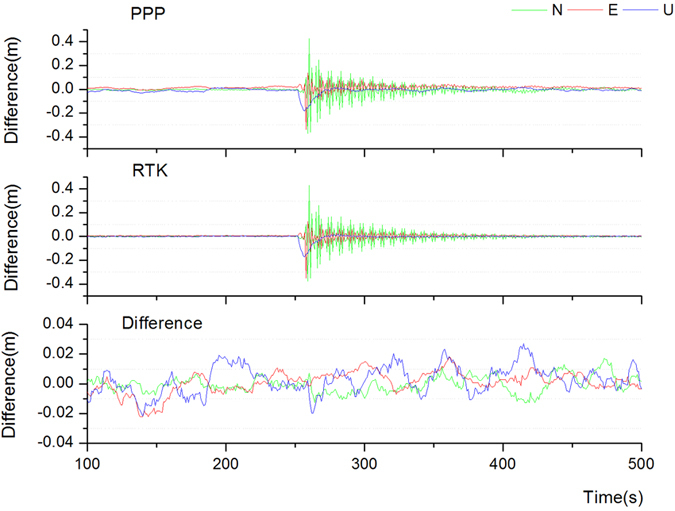
Earthquake simulation experiment results from the vibration platform.

In order to efficiently analyze the effect of the new algorithm for coseismic deformation monitoring, an earthquake simulation experiment was designed, using the earthquake simulation platform located in Wuhan University. For better performance of a shock state, the observation data was collected at a sampling rate of 1Hz. Firstly, for the initial testing phase, a period of convergence time (about half an hour) was completed. Afterwards, the platform was shaken artificially to obtain the vibration time series. For analysis and comparison, we set up another receiver as a reference station, approximately 10 m away from the simulation platform, and the two stations built a ultra short baseline. Then the data was processed by two different strategies: 1. using the new PPP algorithm; 2. using the high precision Power Network software to process the dynamic baseline, the accuracy of RTK calculation could achieve mm level due to the short distance and be considered as the truth-value. Figure 3 showed the comparative analysis of PPP and RTK results.

In the experiment, firstly the spring plate was pressed down to generate damping vibrations in the vertical direction, and then wiggled in the planar direction. As can be seen in Fig. 3, the antenna started to move down at the beginning of the experiment, and then shook constantly in the horizontal direction. Both PPP and RTK results were quite consistent with the actual situation, representing the state of the antenna. The bottom of Fig. 3 illustrated the differences between the PPP and RTK results. The differences for three directions were generally less than 2 cm; and the STD was 0.5 cm, 0.7 cm, 0.9 cm for the N, E and U direction, respectively. The results demonstrated the validity of the new PPP algorithm in simulation coseismic experiment.

### Wenchuan Earthquake Experiment

**Figure 4:**
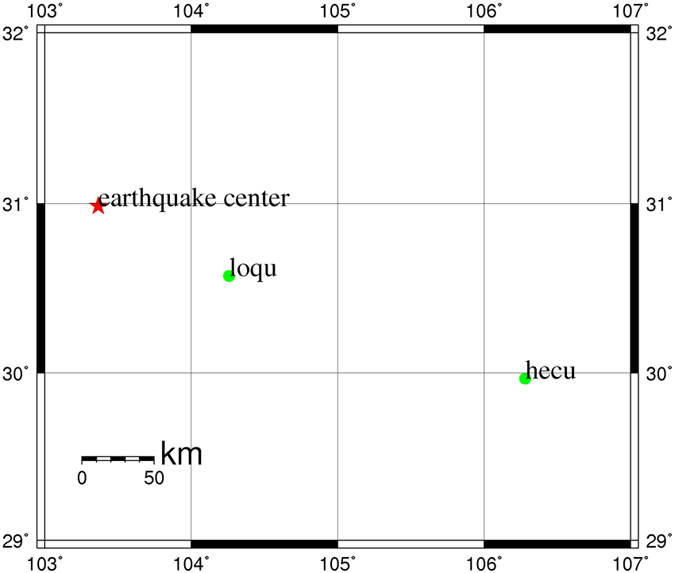
Distribution of the epicenter and stations.

An 8.0 magnitude earthquake occurred at Wenchuan (30.986°N, 103.364°E) in China at 14:28 on May 12, 2008^20^. The observational data from the loqu and hecu CORS stations were processed to analyze the seismic deformation using the new PPP algorithm. The station distribution was shown in Fig. 4. Loqu station was about 100 km away from the epicenter with a 15 s data sampling interval, and hecu station was about 310 km away with a 1 s data sampling interval. Final precise ephemeris and 5 s clock products provided by CODE were used to correct the satellite orbit and clock error during the experiment.

**Figure 5:**
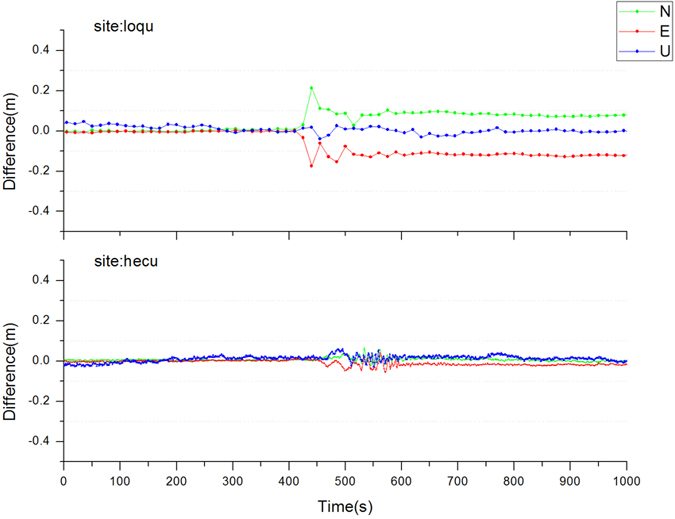
Earthquake displacements between the loqu and hecu stations.

In order to illustrate the displacement movement more clearly, the 1000s results before and after earthquake were intercepted as shown in Fig. 5.

As Fig. 5 showed, loqu station emerged the greatest displacements in the planar direction because of the earthquake. For the north-south direction, the maximum of the displacement was about 22 cm, and eventually remained 8 cm to the north direction; for the east-west direction, the maximum of displacement was about 20 cm and eventually remained 12 cm to the west direction. For the hecu station, because the relatively long distance from the epicenter, the earthquake caused mainly small vibrations and the permanent displacements were only approximately 1 cm in the east and vertical direction. These results agreed well with the day’s solutions before and after the earthquake.

Because of the distance difference between loqu and hecu station to the epicenter, it can also be seen from Fig. 5, the seismic waves successively arrived at the two stations with a time difference of 50 s, which indirectly reflected the seismic wave propagation theory. It can be predicted that based on the gradually improved GNSS observation networks and more precise real-time satellite products, it is feasible to monitor earthquakes using the new PPP algorithm to provide earthquake early-warning alerts.

## Conclusion

In this paper a new PPP algorithm based on the sliding window was presented to improve PPP precision for the coseismic deformation monitoring. Through the simulation dynamic experiment, the new algorithm was proven to be cable to improve positioning accuracy effectively, especially for the elevation direction. To further analyze the effect in earthquake deformation monitoring, a simulation earthquake experiment was carried out using an earthquake simulation platform, and the results showed that the new algorithm could effectively detect the location change of an antenna during an earthquake. The Wenchuan earthquake experiment showed that the new algorithm could be feasible for earthquake early-warning. However, the current experimental analyses were all based on post observational data. Next, we will carry out further research with the real-time data, ephemeris and clock products to realize real-time seismic deformation monitoring and early warning^21^.

## Additional Information

**How to cite this article**: Song, W. *et al.* PPP Sliding Window Algorithm and Its Application in Deformation Monitoring. *Sci. Rep.* **6**, 26497; doi: 10.1038/srep26497 (2016).

## Figures and Tables

**Table 1:** STD of DoY 007, 2012 (unit: m).

	N	E	U
New algorithm	0.008	0.011	0.014
Traditional algorithm	0.013	0.018	0.048
